# Assessment of Social Support and Its Association to Depression, Self-Perceived Health and Chronic Diseases in Elderly Individuals Residing in an Area of Poverty and Social Vulnerability in Rio de Janeiro City, Brazil

**DOI:** 10.1371/journal.pone.0071712

**Published:** 2013-08-12

**Authors:** Valeria T. S. Lino, Margareth C. Portela, Luiz A. B. Camacho, Soraya Atie, Maria J. B. Lima

**Affiliations:** National Public Health School, Oswaldo Cruz Foundation, Rio de Janeiro, Brazil; University of Washington, United States of America

## Abstract

**Objectives:**

Social support (SS) influences the elderly ability to cope with the losses of ageing process. This study was aimed at assessing SS among elderly users of a primary healthcare unit in a poor and violent area of Rio de Janeiro City, and at verifying its association with depression, self-perceived health (SPH), marital status and chronic illnesses.

**Methods:**

A cross-sectional study was performed based on a convenience sample of 180 individuals aged 60 years or older. SS was measured with part of the Brazilian version of Medical Outcomes Study's SS scale, and SPH and depression were assessed, respectively, through one question and the Brazilian version of the Structured Clinical Interview for DSM-IV Axis I Disorders. SS medians were calculated for the categories of SPH, depression, marital status and chronic illnesses variables, and differences were evaluated with the Kruskal-Wallis and Mann-Whitney tests. Additionally, Pearson's chi-square test and logistic regression were employed to identify unadjusted and adjusted associations between SS and those variables.

**Results:**

The participant’s mean age was 73 years old, and level of education was 3 years of school education on average. They were predominantly females (73.3%), and non-married (55.0%). Among them, 74.4% perceived their SS as satisfactory, 55.0% perceived their health as good, 27.8% were diagnosed with major depression and 83.3% had hypertension. Especially for those depressed and with bad SPH, the medians of SS measure were much lower than for others, reaching an unsatisfactory level. Moreover, controlling for other factors, non-depressed individuals were more likely (OR = 2.32) to have satisfactory SS.

**Conclusion:**

in the violent and poor area explored in this research low SS is highly prevalent in the elderly. Depressed individuals are more likely to have low SS and this condition should be investigated in depressed elderly. The reduced scale is useful for low education individuals.

## Introduction

Social networks enable individuals to meet their basic emotional needs for social integration, improving their self-esteem and intimacy with other individuals, through an interactive process of social support (SS) [1]. The concept of social support, in turn, includes the structure and function appropriateness of the social network, but, above all, the degree of an individual's satisfaction with the support he/she benefits from others. Therefore, the difference between network and SS is that the first refers to an individual’s set of relationships, whereas the second focuses on the quality of these interactions [2].

Interpersonal relationships contribute to a greater sense of personal control and offer protection against the effects of stressful events by providing resources, better access to the health sector and habit control, such as alcohol and tobacco consumption [3]–[5].

Social support has a strong impact in older individual’s health and may be crucial in later life, representing a risk factor for many diseases and for mortality, comparable to other well-established risk factors. This has been demonstrated recently in a systematic review and meta-analysis performed by Holt-Lunstad *et al* in order to determine the extent to which social relationships influence mortality risk and which aspects of them are most predictive of mortality. The researchers concluded that comparing people with stronger and weaker social relationships, the first group had a 50% increased likelihood of survival than the second one. Complex measures of SS were more predictive of the risk of death than simple evaluations such as marital status [6].

In the elderly social and medical factors have a complex inter-play that affects important health outcomes. SS from offspring is positively associated with a higher degree of well-being, and less distress and cognitive impairments among older people without a spouse [7]. And the quality of life of low-income women and disability can be positively influenced by the presence of informal support [8], [9].

SS has been incorporated to the concept of social vulnerability, related to deficit accumulation of social factors, that include the sense of life control and coherence, social support, social networks, social engagement, socioeconomic status among other concepts. Andrew *et al* verified that greater social vulnerability is associated with mortality in older adults, especially in the very old [10].

Satisfactory relationships affects physiological responses to stress, decreasing the activation of the autonomic nervous system and hypothalamic-pituitary-adrenal axis, associated with an increase of blood pressure, obesity, hypercortisolemia and dyslipidemia [11]. In the absence of a welcoming social environment those systems’ reactivity increases and there might be harmful consequences for health and longevity, affecting the occurrence of some diseases [12].

One of the leading causes of death globally is coronary artery disease (CAD). SS is a robust predictor of the disease development and progression [13]. Indicators of social isolation are independently associated with elevated risk for the presence of CAD, even after adjustment for age, sex, systolic blood pressure [14]. This evidence points out SS as a promising focus for preventive interventions related to CAD.

Diabetes mellitus 2 (DM2) brings serious negative cardiovascular and neurological consequences representing a burden to society. The association of SS with DM2 may be explained by the fact that SS provides emotional support and information about the disease management, altering the perceived stress and physical reactions which the patient endures [15]. A major task of diabetes care providers is to support patients in performing necessary self-care behaviors using well-accepted strategies [16].

Another disease related to SS is depression, which prevalence achieves 37% in primary healthcare services [17]. This condition is associated to a decrease in quality of life, a greater number of co-morbidity and high mortality rates. Social support protects the individual from the devastating consequences of depression by mitigating the negative effects of psychosocial stressors. There is an inverse relationship between the occurrence of depressive symptoms and the size of social networks [18].

SS is related to other aspects that influence health, as self-perceived health (SPH) and marital status. Elderly individuals with better (SPH) report greater SS [4], [19]. Considering that SPH is a valuable predictor of functional decline [20], institutionalization [21] and mortality [19], strategies aimed at promoting good SPH can lead to a better health control. With regard to marital status, the relationship between this situation and SS is well established. Married people report less stressful events and are more likely to have social contact more often than single people [4], [22], [23].

The availability of more than 20 instruments, with different perspectives and questionable psychometric properties has made difficult the assessment of SS. However, more recently, the definition of the dimensions that composes the SS support construct enabled investigators to associate SS interventions to outcomes and compare different studies [24]–[25]. The following dimensions can be identified: material support, which refers to help given to people in times of need with the provision of access to services, and material resources, including financial help; affective support, which involves expressions of love and affection; emotional support, pertaining to empathy, trust, esteem and interest; information support, regarding an individual's access to counseling, suggestions and guidance; and positive social interaction, referring to the availability of people with whom to have fun and relax [22].

The Social Support Survey of the *Medical Outcomes Study* (MOS) is an instrument that covers the five SS dimensions mentioned previously. It has been validated in Brazil and has also shown its applicability among elderly women of low education levels [18].^ `.^

This study was justified by the understanding of SS as a health conditioning factor, with important implications for the elderly health and health-related quality of life. It was aimed at assessing SS among elderly users of a primary healthcare unit in a poor and violent area of Rio de Janeiro City, and at verifying its association with depression, self-perceived health (SPH), marital status and chronic illnesses.

## Methods

### Study Setting and Sample

A cross-sectional study with a convenience sampling was carried out at the Germano Sinval Faria Teaching Primary Healthcare Center (CSEGSF), at the National School of Public Health, Oswaldo Cruz Foundation, which provides healthcare to residents of Manguinhos district in Rio de Janeiro City. In this region, where the domicile density is 2.8 persons per house, reside more than 31.5 thousand people. The average level of education doesn’t overcome the fundamental in more than 50% of the residents. The houses have only one room and the mean family income is about U$ 300 a month [26]. In the area initiatives targeting a decrease in violence have been facing obstacles due to the activity of drug traffickers who operate there [27].

The non-probabilistic sample comprised individuals selected for their accessibility to the researchers. Elderly individuals, CSEGSF users of 60 years of age or older, were referred, at the researchers’ request, to the geriatric outpatient clinic by physicians and nurses of the Family Health Strategy or by general practitioners of the healthcare center.

This was an exploratory study of the association with main characteristics of the elderly and the sample size was calculated using the prevalence of the most common health problems in elderly individuals, such as dementia and depression, estimated at 20% [17], [28]. The level of significance was established at 5% and the confidence interval at 95%. By using the software Winpepi 2009, version 9.2, the calculated number of volunteers was 180.

### Procedure

This work is part of a research in which a global geriatric assessment was done in elderly individuals, aiming at constructing a screening instrument to the primary care level. The items assessed with traditional geriatric tools were: vision, hearing, grip strength, urinary continence, self- perceived health, mood, cognition, mobility, social support, Body Mass Index, activities of daily living, instrumental activities of daily living, and falls. The SS scale application occurred at the end of the geriatric evaluation, taking approximately five minutes.

Data collection occurred in the period from June to December 2010, during a single consultation. The relevant aspects for this study were the self-perceived health and mood, in addition to social support. Records regarding the diagnoses of hypertension, diabetes, osteoarthritis, and coronary artery disease, as well as sociodemographic data like age, sex, level of education and marital status, were collected from patient charts, considering their potential to influence SS [4], [22], [23], [29].

The volunteers included in the study were those who agreed to take part in the research by signing the informed voluntary consent form and who were capable of understanding the survey questions. The ones with dementia, severe visual or hearing impairments were excluded after cognition, vision and hearing assessment during geriatric evaluation.

Self-perceived health was assessed through a five-point Likert scale – very good, good, not very good, bad or very bad – applied to one question largely employed with that objective: “When you compare yourself with other people of your age, how do you feel is your health currently?” [30].

For the diagnosis of major depression, we applied the Structured Clinical Interview for DSM-IV Axis I Disorders – Clinician Version, that has been widely employed, and it has proven to be a useful instrument for that condition diagnosis. It follows an interview script and targets the application in clinical practice. Its test-retest reliability has been verified in Brazil with good results for depression (Kappa = 0.87) [31].

SS measurement was made with part of the Brazilian version of The Social Support Survey of the *Medical Outcomes Study* (MOS) [4]. MOS is an instrument validated in a study with over 3,000 chronic patients followed by different health care services in the United States. It covers the five SS dimensions and presents 19 questions related to the availability of people to satisfy the needs of each dimension. There are five possible answers subject to scoring: 1 (never); 2 (once or twice); 3 (a few times); 4 (fairly often) and 5 (very often) [22]. In Brazil the scale’s Portuguese version has shown good internal consistency, stability and construct validity and it has been deemed appropriate for use in studies on the the association between SS and outcomes related to health [4], [32]. The SS dimensions presented internal consistency varying between 0.75–0.91, and 0.86–0.93 in the test and retest, respectively [4].

Reducing the instrument made SS level assessment possible together with the other functions in a single consult. Considering the importance of material and emotional support and of social interaction in the lives of elderly individuals, such dimensions were selected to be included in a wide geriatric evaluation. This strategy is characterized by a multidimensional approach where the goal is to examine an individual’s different functions.

The use of a scale with those three dimensions was also based on the verification of an association between affective support and positive social interaction; and emotional and informative support, through a factor analysis in the Brazilian study of MOS validation, in which the Cronbach’s Alpha was equal or superior to 0.83 for all dimensions [4].

The adapted scale contained four questions for each type of support totaling 12 questions, with answers ranging from never (1 point) to always (5 points). The sum of all answers determined the SS score, which ranged from 12 to 60 points (annex 1). It was verified that elderly individuals showed better understanding when the initial sentence was divided into two questions. Thus, initially elderly individuals were asked about the existence of someone they could rely on in different situations and then they were asked about the frequency of support.

### Data Analysis

The variable targeted in this study was social support, categorized as satisfactory for a score of 48 or above, since such individuals would already have support almost always (4 points) or always (5 points) in all situations presented, and unsatisfactory SS for a score of 47 or below.

We aimed to identify the association of SS with: self-perceived health - answers grouped in the categories good (very good/good), not very good and bad (very bad/bad); depression; marital status (married/cohabiting and single/widow/widower/divorced); and the chronic illnesses CAD, hypertension, diabetes (DM2) and osteoarthritis.

SS medians were calculated according to the categories: SPH, depression, marital status and chronic illnesses, in order to evaluate the distribution of satisfactory and unsatisfactory SS according to those variables.

The statistical significance of the differences between SS and SPH medians was analyzed with the Kruskal-Wallis test for non-parametric samples due to the three possible answer categories; and between SS and the other variables, which allowed only two types of answer, the Mann-Whitney test was used.

Pearson's chi-square was employed to verify the association of SS with marital status, depression, osteoarthritis and diabetes. Since SPH was expressed in terms of an ordinal three-category variable, it was also verified the possibility of SS increase with SPH improvement by linear trend chi-square.

Through logistic regression with multivariate analysis we verified the association between SS and depression, SPH, marital status and chronic illnesses, adjusting the simultaneous effects of multiple co-variables. Covariates were entered in a stepwise forward strategy, and the final model considered those achieving statistical significance (p<0.05) or found to be more relevant for the explanatory model.

The software used in the analyses was SPSS for Windows (Statistical Package for the Social Sciences), version 18.

### Ethics Statement

The research was approved by the Research Ethics Committee of Sergio Arouca National School of Public Health at Oswaldo Cruz Foundation (FIOCRUZ). According to report number 126/10. All participants signed the informed voluntary consent, in which privacy and anonymity of participants was assured.

## Results

One hundred and eighty five individuals were referred to the research; five were dismissed due to severe visual impairment and advanced dementia. During the conduction of an SS survey one person was unable to understand the questions and was not included in the analyses. The study participants show a mean age of 73 years old. The group was predominantly of the female sex and had low education levels. There was a slightly greater portion of single individuals and widows/widowers. Despite 74.4% perceiving their SS level as satisfactory and assessing their health as good or not very good, nearly a third of the elderly individuals (27.8%) were diagnosed with major depression. With respect to chronic illnesses, 83.3% and 31.7% had hypertension and diabetes, respectively, demonstrating high prevalence of both diseases. Descriptive statistics are presented in Table 1.

**Table 1:** Characteristics of study participants (n = 180).

Variable	Mean	SD
Age	73	[6.9]
Level of Education (years)	3	[2.9]
	**n**	**%**
Sex		
Female	132	73.3
Male	48	26.7
Marital status		
Married	81	45.0
Single/widow(er)	99	55.0
Chronic illnesses		
Hypertension	150	83.3
Diabetes	57	31.7
CAD	18	10.0
Osteoarthritis	77	42.8
Social Support		
Satisfactory	134	74.4
Unsatisfactory	45	25.0
Ignored	1	0.6
SPH		
Good	99	55.0
Not very good	60	33.3
Bad	20	11.1
Ignored	1	0.6
Depression		
Yes	52	28.9
No	127	70.5
Ignored	1	0.6

SPH, self-perceived health; CAD, coronary artery disease;

SD, standard deviation.

The distribution of the scores obtained for the variable SS shows higher values of satisfactory SS for those non-depressed (76.7%) and those with good (58.6%) or not very good (33.1%) SPH. There was a significant difference between the groups. For the other variables, although there was a significant difference in the distributions of the two SS categories into diabetes, osteoarthritis and marital status, the cut-off point of 48 for satisfactory SS was not observed (Table 2).

**Table 2:** Social support medians across categories of the variables depression, self-perceived health, marital status and chronic illnesses (n = 179).

Variable	n	SS Median (min-max)	*p*
Depression			<0.001^1^
No	127	60 (20–60)	
Yes	52	48 (23–60)	
Self-perceived health			0.003^2^
Good	99	60 (24–60)	
Not very good	60	59 (20–60)	
Bad	20	48 (23–60)	
Marital status			
Married	81	60 (20–60)	0.014^1^
Single/widow(er)	98	54 (23–60)	
Diabetes			
No	122	57 (20–60)	0.048^1^
Yes	57	60 (24–60)	
CAD			
No	161	58 (20–60)	0.649^1^
Yes	18	58.5 (29–60)	
Hypertension			
No	30	56 (28–60)	0.424^1^
Yes	149	59 (20–60)	
Osteoarthritis			
No	102	60 (27–60)	0.009^1^
Yes	77	54 (20–60)	

1 Mann-Whitney Test;

2 Kruskal-Wallis Test; CAD- coronary artery disease; significance level set for the analyses – p value <0.05.

The association between SS and mood disorder became evident in the multivariate analysis. Non-depressed individuals were more likely (OR = 2.32) to have satisfactory SS, controlling for other factors. However, the association of SS with SPH, marital status and osteoarthritis disappeared, revealing the presence of depression as a confounding factor. Chronic illnesses were not associated with SS and were not included in the final model to keep it parsimonious (Table 3).

**Table 3:** Association between Social Support (satisfactory vs. unsatisfactory) and depression, self-perceived health, marital status and chronic illnesses (n = 179).

Variable	Satisfactory SS	Unsatisfactory SS	Bivariate analysis		Multivariate analysis	
	n (%)	n (%)	*p*- value	crude OR (CI)	*p*- value	adjusted OR (CI)
SPH						
VG/Good	78 (58.6%)	21 (21.2%)	0.039^1^	3.04 (1.11–8.30)	0.39	1.88 (0.62–5.68)
Not very good	44 (33.1%)	16 (26.7%)		2.25 (0.78–6.43)		1.61 (0.53–4.91)
VB/Bad	11 (11.0%)	9 (45.0%)		1.00		1.00
Depression						
No	102 (76.7%)	25 (54.3%)	0.005^2^	2.76 (1.36–5.59	0.03	2.32 (1.07–5.02)
Yes	31 (23.3%)	21 (45.7%)		1.00		1.00
Marital status						
Married	66 (49.6%)	15 (32.6%)	0.04^2^	2.03(1.01–4.19)	0.08	1.92 (0.92–3.97)
Single/widow(er)	67 (50.4%)	31 (67.4%)		1.00		1.00
Osteoarthritis						
No	81 (60.9%)	20 (43.5%)	0.04^2^	2.02 (1.02–3.99)		
Yes	52 (39.1%)	26 (56.5%)		1.00	–	–
Diabetes						
No	89 (66.9%	34 (73.9%)	0.37^2^	1.41 (0.66–2.96)	–	–
Yes	44 (33.1%)	12 (26.1%)		1.00		
Hypertension						
No	21 (15.8%)	8 (17.4%)	0.79^2^	0.89 (0.36–2.17)	–	–
Yes	112 (84.2%)	38 (82.6%)		1.00		
CAD						
No	121 (91%)	40 (87.0%)	0.43^2^	1.52 (0.53–4.29)		
Yes	12 (9.0%)	6 (13.0%)		1.00	–	–

SPH, self-perceived health; CAD, coronary artery disease; CI, Confidence Interval; VG, very good; VB, very bad; OR, *Odds Ratio*;

1 Linear trend chi-square;

2 Chi-square; significance level set for the analyses – p value <0.05.

## Discussion

The elderly in this study predominantly (74.4%) presented satisfactory SS, result that is similar to that of other studies involving the same age group, in different socioeconomic contexts [18], [33]. This study also supports the idea of reducing dimensions of the original SS construct in the MOS [27], [37]–[39], once the use of questions pertaining to only three dimensions does not seem to have affected the assessment of SS to elderly individuals, considering the strong association between unsatisfactory SS and depression. The advantage of the instrument reduction was felt in the context of the primary healthcare visits, in which the duration of attendance by a doctor is usually short. Professionals and elderly found it easy to use and understand, respectively.

Additionally, the choice for using a cut-off point to categorize SS into satisfactory or unsatisfactory, similarly to the strategy applied to discriminate patients with the Social Support Inventory by Enhancing Recovery in Coronary Heart Disease (ENRICHD), seemed appropriate, generating a pragmatic way of defining the need or not of interventions [40].

### Relationship between SS and Depression

The multivariate analysis demonstrated the association between SS and mood disorder. Non-depressed individuals were more likely (OR = 2.32) to have satisfactory SS. The average SS scores observed in this study are consistent with others which demonstrate a lower likelihood of depression among people with high levels of SS when compared to those with low SS. In Korea, using a local version of MOS, Shin *et *al [38] diagnosed depression in the group with low SS three times as much as in the group with high SS, while in Thailand the association between depression and functional incapacity was modified by the level of SS offered [39]. Suicidal ideas, common in individuals with severe depression, occurred more often in Australian elderly individuals with precarious SS [41].

SS acts like a buffer protecting individuals against the development of depression, especially when they are exposed to stressors, like negative life events [42]. This effect becomes important in regions like Manguinhos where the elderly are exposed to violence as part of everyday life.

Sonnenberg et al studied the gender differences in the relation between depression and SS in a population based sample aged 55–85 years, with 2,823 people at baseline and using the 13-year follow-up data on onset of depression. Like our study, they verified that respondents with low SS were more often depressed but men showed higher rates of depression than women [43].

Melchiorre et al investigated the associations between SS, demographics/socio-economics, health variables and elder mistreatment in a cross-sectional study with 4,467 not demented individuals aged 60–84 years living in seven European countries. Like our work they verified that low scores on depression were indicators of high SS. In this study, they also found association between low levels of SS and abuse, particularly psychological abuse [44].

Overall, the studies point to the existence of a clear association between depression and low SS. Considering this aspect as a modifiable risk factor, conducting studies focusing on preventing SS decline or improving it favor a decrease in depression-related symptoms and maintaining the mental health of elderly individuals.

### Relationship between SS and SPH

The association between SPH and SS, as shown by bivariate analyses, was attenuated when the multivariate analysis was carried out, which demonstrated the strong influence of depression as a confounding factor in those patients, which is consistent with the association of depression and SPH reported in previous studies [45], [46]. Even so, SPH is considered an independent risk factor both for morbidity and mortality. This occurs even when other clinical and psychosocial risk factors are controlled [47] and reflects the individual’s integrated perception of the biological, psychological and social dimensions of health [48].

In this investigation, 55% of people reported good or very good SPH, a rate that is much lower than that observed in the general population of European countries, especially the most developed ones, where it can be higher than 80% [33]. This is not surprising not only due to the focus on elderly individuals [49], but also due to the unfavorable environmental and social characteristics of the community considered.

The association between SS and SPH has already been reported in different national and international studies [50]–[52]. Lima-Costa *et al* verified that elderly individuals are two to four times more likely to rate their health as poor when they were unhappy with their social network, especially in the most underprivileged groups [50]. In another research, lower MOS scores were associated with higher chances of bad SPH among women with lower income [51]. And in a sample of over 5,000 Australians, both the size of the social network and the level of satisfaction with the support they received were associated with SPH [52].

The disappearance of the association between SPH and SS in the multivariate analysis demonstrated the strong influence of depression as a confounding factor. A possible explanation for this result is the size of the sample, which may have been insufficient. Even so, this finding suggests that a mood disorder control may be necessary when analyzing the association between SPH and SS.

### Relationship between SS, Marital Status and Chronic Illnesses

Although the relationship between marital status and SS has been reported by different authors [4], [22], [23], [53], it also disappeared, in this study, when the multivariate analysis was carried out. Depression was, again, a confounding factor.

On the whole chronic illnesses were not significantly associated with SS. With regard to diabetes and hypertension most studies which report this association focus on disease control [54]–[57]. Since our study did not address aspects pertaining to the management of those conditions, it is possible that this fact has been decisive to the lack of association between SS, diabetes and hypertension.

In relation to osteoarthritis, the association with SS was largely due to the presence of depression. A marginal significance remained after adjustment. Since the most prominent symptoms of that disease are pain and physical disability, both decisive factors to declining quality of life, the occurrence of depression among those patients is not surprising. SS may reduce stress and mitigate the negative effects of the disease, including depression, resulting in greater satisfaction with life and better ability to cope with osteoarthritis. Luger *et al* used the MOS and demonstrated that SS was related to well-being in patients with osteoarthritis [58]. They also suggested the increase in social relationships as part of the therapeutic strategy.

Although evidence suggests that low SS confers a risk of 1.5 to 2.0 in the development and progression of CAD in both healthy populations and in patients with the established disease [59], the expected association between SS and CAD was not found in this research (*p* = 0.649). The probable cause for this finding is that the prevalence of the disease was underestimated in our sample. There were only 18 charts (10%) which recorded the condition and this possibly reflects smaller access to complementary diagnostic methods available in that primary care service.

This study presents some limitations. The insufficient size of the sample may have limited the demonstration of association between SS, diseases and other conditions that affect health. Aspects concerning the management of illnesses which contribute to the association between SS and DM2 have not addressed. We also could not establish a causal link between depression and low SS. Finally, this research has been carried out in a healthcare unit with a greater proportion of women in the sample. These considerations limit the results generalization.

### Conclusion

We conclude that in the violent and poor area where this research was developed, low SS is highly prevalent among the elderly, and depressed individuals are more likely to have unsatisfactory SS. Low SS should be investigated in depressed elderly, making it possible to carry out group interventions to promote health and well-being.

The use of instruments which are easy to apply enables the healthcare team to diagnose and intervene in a timely manner through the organization of support networks and individualized action, and it may result in positive outcomes in life conditions of elderly individuals.

The reduced MOS social support scale is a useful instrument for the assessment of low education elderly in the primary healthcare environment. The adoption of a cut-off point of 48 may facilitate the diagnosis of an individual’s level of support and promote more agile decision-making during a medical visit.
